# Adolescent Psychopathology and Cognitive/Academic Functioning: Impact of Comorbidity Using a Genetically Sensitive Design

**DOI:** 10.1192/bjo.2024.221

**Published:** 2024-08-01

**Authors:** Olakunle Oginni, Fruhling Rijsdijk, Adam Tarnoki, David Tarnoki, Kaili Rimfeld

**Affiliations:** 1 Cardiff University, Cardiff, United Kingdom; 2 Anton de Kom University of Suriname, Paramaribo, Suriname; 3 King's College London, London, United Kingdom; 4 Semmelweis University, Budapest, Hungary; 5 Royal Holloway University of London, London, United Kingdom

## Abstract

**Aims:**

To determine: i. the nature of the associations between three domains of psychopathology (depressive, hyperactivity and conduct symptoms) and cognitive/academic performance among adolescents i.e., whether these reflect causal processes and/or common genetic effects; ii. The extent to which these associations vary by comorbidity.

**Methods:**

The sample comprised participants in the UK Twins Early Development Study (TEDS; n≈12,000 individuals) assessed for depressive, hyperactivity and conduct symptoms using standardised questionnaires. Cognitive and academic performance were assessed using Standard Progressive Matrices and GCSE scores respectively. Comorbidity was derived as a count of borderline/high psychopathology scores present per individual. Twin modelling was used to investigate preliminary correlations and moderation effects. Genetic models were further used to determine the most likely direction of causal effects with/without genetic correlations.

**Results:**

There were small to moderate negative correlations between adolescent psychopathology domains and cognitive performance (−0.01 ≤ *r*≤−0.15) and academic performance (−0.06 ≤ *r*≤−0.23). Correlations were smallest for depressive symptoms and larger for hyperactivity/conduct symptoms. The correlation between hyperactivity symptoms and cognitive performance was significantly more negative as comorbidities increased (moderation coefficient – β_mod_ = 0.07, 95% CI: 0.02, 0.12). Similarly, the association between depressive symptoms and academic performance also became more negative as comorbidities increased (β_mod_ = −0.08, 95% CI: −0.11, −0.05). Twin modelling indicated that hyperactivity symptoms were causally associated with poorer cognitive and academic performance. In contrast, poorer cognitive performance was causally associated with conduct symptoms.

**Conclusion:**

These preliminary findings indicate the impact of comorbidity on the functioning of adolescents with hyperactivity and depressive symptoms. They further suggest the need to specifically recognise these comorbidities during assessment and treatment planning to promote optimal functioning. Our findings also suggest differential mechanisms for the links between different psychopathology domains and impaired functioning. Further analyses will investigate moderation of the causal links and/or genetic correlations and whether these associations vary by indicators of marginalisation (sex and ethnicity).

